# Superior mesenteric artery syndrome and anorexia nervosa: a case report

**DOI:** 10.1186/s13256-023-04168-6

**Published:** 2023-11-04

**Authors:** Shivani Singh, Ann L. Contrucci

**Affiliations:** Philadelphia College of Osteopathic Medicine, Suwanee, GA USA

**Keywords:** Anorexia nervosa, Eating disorder, Superior mesenteric artery syndrome, Duodenal obstruction, Aortomesenteric angle

## Abstract

**Background:**

Superior mesenteric artery (SMA) syndrome is an underdiagnosed complication in anorexia nervosa (AN) patients, which results from weight loss-induced atrophy of the mesenteric fat pad, causing compression of the third part of the duodenum. SMA syndrome can be life-threatening as its nonspecific symptomatology often results in a delayed diagnosis. It is a rare condition, but its true prevalence may be higher than the reported numbers. A history of persistent nausea and vomiting after oral intake and weight loss in AN should raise suspicion about this diagnosis, as weight loss is the most significant factor in this diagnosis. Other high-risk factors include rapid, extreme weight loss, anatomical abnormalities, or a history of prior abdominal or spinal surgeries.

**Case presentation:**

The patient presented in this report was a 26-year-old Caucasian female with a history of severe enduring anorexia nervosa. This patient suffered from an insidious case of SMA syndrome secondary to AN. This patient presented with vague symptoms of nausea and vomiting, persistent abdominal pain, and rapid weight loss. The patient was successfully treated but could have had a much different outcome if the diagnosis had been further delayed.

**Conclusions:**

An awareness of SMA syndrome and its clinical presentation within similar populations can prevent complications and even fatalities that come with it.

## Introduction

Anorexia nervosa (AN) is characterized by self-starvation, malnutrition, excessive weight loss in children, adolescents, or adults, or lack of proper weight gain in growing children, due to an intense fear of weight gain or a distorted body image [1]. According to the DSM-5, in order to be diagnosed with anorexia nervosa, three criteria must be met:

1. Restriction of energy intake relative to requirements, leading to significantly low body weight in the context of age, sex, developmental trajectory, and physical health (less than minimally normal/expected).
2. Intense fear of gaining weight or persistent behavior that interferes with weight gain.
3. Disturbed by one’s body weight or shape, self-worth influenced by body weight or shape, or persistent lack of recognition of the seriousness of low body weight.

Despite its seemingly psychiatric etiology, AN is a complex medical disease with neurobiological and metabolic implications. The DSM-5 characterizes AN into two different subtypes—the restricting type and the binge-eating/purging type [1]. The restricting type is defined as an individual in which weight loss is accomplished through dieting, fasting, and/or excessive exercise. Those with the restricting subtype have not engaged in binge eating or purging behaviors during the last three months [1]. The binge-eating/purging type presents in a patient that has engaged in recurrent episodes of binge-eating or purging within the last three months, including behaviors such as self-induced vomiting, misuse of laxatives, diuretics, or enemas [1].

While AN primarily affects adolescent girls and young women, approximately 10% of patients are males [2]. Nearly 30 million individuals in the United States have been diagnosed with an eating disorder, making up approximately 9% of the US population [2]. There are approximately 10,200 deaths yearly in the US due to eating disorders, accounting for 1 death every 52 min [2]. Anorexia nervosa is the only psychiatric illness with significant and often deadly somatic complications, affecting every single organ system of the body, which get worse as the disease progresses [3]. Due to its vast somatic complications, AN has the highest mortality rate of all eating disorders [3].

Although there is very little research on SMA syndrome in AN, there should be more parity in diagnosis and recognition between medical illnesses and psychiatric illnesses. This case report depicts the unique distinction that anorexia has by being the only psychiatric disease with multiple, often life-threatening, medical complications. Normally, the mesenteric fat pad tethers the superior mesenteric artery and prevents movement. SMA syndrome is a disease resulting from a loss of the mesenteric fat pad around the SMA, causing the compression of a portion of the duodenum as it passes between the abdominal aorta and the SMA [4]. As noted previously, many of the symptoms of SMA syndrome can also be attributable to anorexia nervosa [3, 4]. Therefore, a physician must have a high index of suspicion to make this generally rare diagnosis. It should be considered in patients with significant weight loss, early satiety, and persistent abdominal pain in the setting of the diagnosis of anorexia nervosa.

## Case presentation

The patient was a 26-year-old Caucasian female with a history of severe enduring anorexia nervosa. She was diagnosed with anorexia nervosa, restrictive subtype, at age 16, although symptoms began at age 13. She entered her first residential treatment at age 18, with multiple admissions for a total of 6 from ages 18–26. She had also been hospitalized for acute medical stabilization 7 times since age 15. The patient’s height was 5′5.5″. Her highest weight was 127 pounds at age 13–127 pounds with a Body Mass Index (BMI) of 20.8; her lowest at age 19–82 pounds with a BMI 13.4. She returned to residential treatment at age 26 for 2 months, and almost immediately after discharge, lost 17 pounds over the course of the next 2 months, going from a weight of 116 pounds (BMI 19) to 99 pounds (BMI 16.2). At that time, she was seen initially in the emergency department for the chief complaint of “unintentional weight loss and nausea with a history of anorexia nervosa” but was discharged home with the diagnosis of “non-intractable vomiting with nausea, unspecified vomiting type” after laboratory studies were determined to be “normal.” She was subsequently seen again three weeks later in the same emergency department for additional complaints including “nausea, vomiting, diarrhea, unintentional weight loss (followed by a dietician), worsening pain, decreased intake.” She was discharged home a second time with the same diagnosis of “non-intractable vomiting with nausea, unspecified vomiting type” after “normal” labs were noted. On her third emergency department visit six days later, she was hospitalized for severe malnutrition and persistent nausea, vomiting, and inability to eat as well as early satiety and constant “fullness.” Of note, her physical exam revealed no abnormalities other than thin body habitus noted, and her lab studies again were “normal.” Her total weight loss at this time over the two-month period was 15.5% of total body weight, with a BMI trending from 19 to 16.2. While placing a DobHoff tube for enteral feedings, it was noted there was difficulty advancing the tube on fluoroscopic guided placement; SMA syndrome was suspected and then confirmed on ultrasound with the findings of “acute aorto-mesenteric angle which can be seen with SMA syndrome.” She was treated for three weeks with nutritional rehabilitation, and her symptoms consistent with SMA syndrome slowly improved with weight gain. Although the patient remained underweight, her SMA syndrome did resolve with consistent weight gain. Specifically, her abdominal pain and early satiety resolved.

## Discussion

SMA syndrome is a rare and uncommon complication of AN, but should be kept in mind as a cause of intestinal partial obstruction. This diagnosis should be suspected in a patient with a history of persistent postprandial abdominal bloating and pain, vomiting, and weight loss [5]. It is imperative to rule out other diagnoses that can cause abdominal pain and obstruction of the duodenum. Regardless of its rarity, the mortality associated with SMA syndrome complications makes it a crucial differential diagnosis when considering bowel obstruction with recent weight loss [6]. Mortality associated with SMA syndrome is due to a failure to diagnose the condition because, if left untreated it can progress to bowel obstruction and perforation [7]. Since the presentation of AN can be similar, an SMA syndrome diagnosis can be delayed or missed with a substantial delay in starting effective treatment, as noted in this case—the patient was seen three times in the emergency department before being admitted to the hospital.

SMA syndrome can result from other medical conditions that lead to weight loss and a malnourished state, in addition to anorexia nervosa [5]. Due to the diminished mesenteric fat, the compression of the SMA against the abdominal aorta causes either a partial or complete blockage which prevents the progression of food or fluid into the rest of the small intestine [5]. SMA syndrome in general is uncommon and the prevalence is unknown, but barium studies have estimated that the worldwide incidence ranges from 0.013 to 0.3%, which translates to 41,000–96,000 individuals in the US [4]. Of these individuals in the US population, there is a reported 1 in 3 mortality rate [4]. Although SMA syndrome can occur at any age, including infants and the elderly, it is reported in greater frequency among teenagers and young adults [8]. SMA syndrome affects more women than men by a 3:2 ratio [9]. There has not been enough research to date to determine the prevalence of SMA syndrome as a complication of eating disorders.

Much of the literature focuses on AN’s psychiatric implications and co-morbid mental illnesses rather than considering it a systemic neurobiological and metabolic disease. In fact, while AN weight loss is one of the most common causes of SMA syndrome, there was little data and statistics found to correlate the two. This case is an example of how the diagnosis of SMA syndrome was overlooked. The patient was seen many times before the diagnosis was made. It is rather easy to be a victim of confirmation bias, where clinicians focused on the “normal labs,” concluding that her symptoms of early satiety, abdominal pain, and emesis were due to her eating disorder only.

Patients with SMA syndrome usually complain of early satiety, nausea, indigestion, and epigastric pain that typically begins 15–20 min after oral food intake and is relieved by vomiting or the passage of a few hours [4]. More severe abdominal pain can occur after food or drink intake due to the SMA pulsating forcibly against the duodenum because of the lack of a mesenteric fat pad [6]. This pain is often followed by food aversion or food fear, another parallel with AN symptoms, which exacerbates weight loss and worsens SMA syndrome [4]. Because of this overlapping presentation with typical AN symptoms, this vague presentation is attributed to complications of AN, before considering SMA syndrome.

**Fig. 1:**
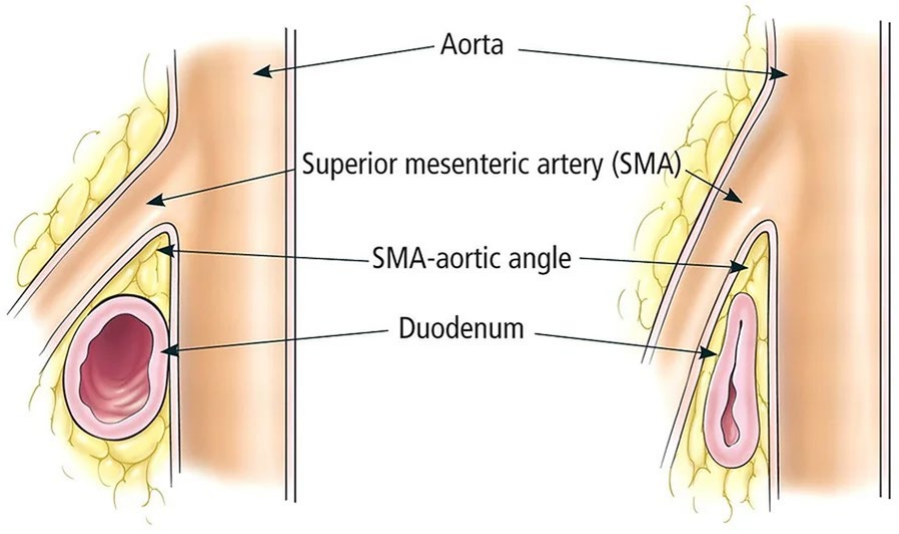
This diagram shows the normal angle between the SMA and aorta on the left and shows the narrowed angle on the right caused by SMA syndrome [10]

Besides significant weight loss, SMA syndrome is most associated with malabsorption syndromes, AIDS, trauma, and burns [9]. Surgical interventions that may distort normal anatomy can lead to this syndrome, including spinal surgery for scoliosis and esophagectomy [9]. In fact, SMA syndrome is more commonly reported as secondary to spinal or gastric bypass surgery, malabsorption syndrome, or more severe illnesses, such as malignancy [4]. Other diseases that may mimic the symptoms of SMA syndrome include duodenal dilation and decreased motility secondary to scleroderma, malrotation, collagen vascular diseases, and mesenteric ischemia [6]. For a thorough evaluation, it is recommended that patients undergo extensive testing to rule out differential diagnoses, including dyspepsia, reflux, and other causes of small bowel obstruction [4]. Diagnosis of SMA syndrome is typically made by an upper gastrointestinal series, abdominal computed tomography (CT), computed tomography angiography (CTA), or Doppler Ultrasound [4]. On radiography, the angle between the abdominal aorta and SMA can be measured, it is normally 25°-60°, but in SMA syndrome, the aortomesenteric angle is reduced to 7°–22° with an aortomesenteric distance less than 8 mm [4]. The schematic in Fig. 1 provides a side by side comparison of a normal and narrowed aortomesenteric angle.

Depending on the severity of the obstruction, the pain can be intermittent or chronic [7]. Acutely, this mechanical obstruction causes dilation of the stomach, which can be life-threatening. With chronic intestinal obstruction, there is a continuous cycle of intermittent post-prandial pain, vomiting, and weight loss, which prolongs the cycle more [7]. This case also illustrates that SMA syndrome can be managed with parenteral feedings and additional nutritional rehabilitation, leading to full recovery. Three weeks after parenteral feeding with a DobHoff tube, the patient’s SMA syndrome and symptoms resolved. Had her condition been overlooked for much longer, the outcome may have been different. This patient embodies a struggle that many patients with eating disorders similarly often encounter.

Most patients with SMA syndrome, regardless of their etiology, are initially treated conservatively with treatment goals including restoration of retroperitoneal fat to decrease symptoms caused by bowel obstruction [4]. The conservative approach includes nutritional support, fluid resuscitation, and correction of electrolyte abnormalities [11]. If the patient fails to respond to conservative therapy, surgery is indicated, including gastrojejunostomy and duodenojejunostomy, which have high success rates [11]. As shown in this patient’s case, accurate diagnosis and treatment took more than one month, but once treated, the recovery was quick, emphasizing how important it is to consider it as a potential diagnosis in certain patients. Much of the literature on AN and other eating disorders heavily focuses on psychiatric implications and co-morbid mental illnesses rather than considering it a systemic neurobiological and metabolic disease. In fact, while AN weight loss is one of the most common causes of SMA syndrome, there was little data and statistics found to correlate the two.

## Conclusion

In conclusion, this case demonstrates that, in the proper clinical setting of a cachectic AN patient suffering from postprandial abdominal pain and nausea, a high index of suspicion should be maintained to diagnose SMA properly. These symptoms should not be attributed to eating disorders alone without properly investigating them. The diagnosis of SMA syndrome must be based on clinical symptomatology correlated with radiographic information. If diagnosed in time, SMA can be reversed, and a potential catastrophe can be avoided. This is a situation where the adage, “when you hear the hoofbeats, think about horses, not zebras,” may not hold true. SMA syndrome poses unique diagnostic challenges, and an awareness of its clinical presentation can further improve patient outcomes and avoid potentially life-threatening complications.

## Data Availability

Data sharing is not applicable to this article as no datasets were generated or analyzed during the current study.

## Abbreviations

AN: Anorexia nervosa
SMA: Superior mesenteric artery
BMI: Body mass index
CT: Computerized tomography
